# Type 1 T Helper Cell-Based Molecular Subtypes and Signature Are Associated with Clinical Outcome in Pancreatic Ductal Adenocarcinoma

**DOI:** 10.3389/fcell.2022.839893

**Published:** 2022-04-01

**Authors:** Ran Wei, Huihui Zhang, Jianzhong Cao, Dailei Qin, Wuguo Deng, Shengping Li

**Affiliations:** ^1^ Collaborative Innovation Center for Cancer Medicine, State Key Laboratory of Oncology in South China, Sun Yat-sen University Cancer Center, Guangzhou, China; ^2^ Engineering Research Center of Cell and Therapeutic Antibody, Ministry of Education, Pharm-X Center, School of Pharmacy, Shanghai Jiao Tong University, Shanghai, China

**Keywords:** pancreatic ductal adenocarcinoma, type 1 T helper cell, lymph node metastasis, immunotherapy, prognosis

## Abstract

Lymph node metastasis in pancreatic ductal adenocarcinoma (PDAC) is shown to be related with poor prognosis. To construct an immune-related gene prognostic risk model for PDAC and clarify the molecular and immune characteristics and the benefit of immune checkpoint inhibitor (ICI) therapy in prognostic risk model-defined subgroups of PDAC, we analyze the association between the density of immune cell infiltration and lymph node metastatic status and further study the potential role of immune cells, immune cell–related genes, and immunotherapy outcomes in PDAC patients using bioinformatics models and machine learning methods. Based on The Cancer Genome Atlas (TCGA), PACA-AU and PACA-CA data sets, 62 immune-related hub genes were identified by weighted gene co-expression network analysis (WGCNA). Four genes were selected to construct a molecular subtype identification based on the type 1 T helper cell–related hub genes by using the Cox regression method. We found that lower type 1 T helper cell abundance was correlated with prolonged survival in PDAC patients. Further, prognostic risk score model constructed with the type 1 T helper cell-related signature showed high accuracy in predicting overall survival and response to immunotherapy. This study might improve the understanding of the role of type 1 T helper cells in PDAC patients and aid in the development of immunotherapy and personalized treatments for these patients.

## Introduction

Pancreatic ductal adenocarcinoma (PDAC) is a common lethal disease owing to its high invasion and metastatic ability (Sung et al., 2021). Despite the prominent clinical importance of metastasis, the metastatic process remains unclear. Cancer cells invade and metastasize mainly into the surrounding tissues, including lymph nodes, nerves, and the retroperitoneal plexus, which causes recurrence after the radical resection of PDAC. Several recent studies report that lymph node metastatic (LNM) status is one of the most important predictors of prognosis in PDAC patients who received curative resection, and it is regarded as a best predictive sign with remarkable clinical significance for risk stratification and therapeutic decision making in these patients (Lowder et al., 2018; Honselmann et al., 2020; Nishiwada et al., 2020).

The tumor grows and develops in a complex microenvironment, containing various immune cells, stromal cells, and some other cell types (Ho et al., 2020). Type 1 T helper (Th1) cells are a subset of T helper cells when naïve CD4^+^ T cells (Th0) are activated by antigen-presenting cells (APCs) in the presence of interleukin (IL)-12, and express the T-box transcription factor TBX21 (T-bet), which produce anticancer factors, including interferon gamma (IFN-γ), IL-2, and tumor necrosis factor α (TNF-α) (Saravia et al., 2019). However, IFN-γ may also have pro-tumorigenic effects as it could increase programmed cell death-ligand 1 (PD-L1) expression in cancer cells, impairing antitumor immunity (Abiko et al., 2015). Thus, the effect of Th1 cells is uncertain in PDAC patients. During recent years, immunotherapy has demonstrated clinical responses in a variety of solid tumors, including melanoma and lung cancer, but results in pancreatic cancer have been disappointing. PDAC seems to be almost entirely refractory to immunotherapy. The special tumor microenvironment of pancreatic tumors may be responsible for this phenomenon. The tumor microenvironment of a pancreatic tumor is characterized by a prominent myeloid cell infiltration typically devoid of CD8^+^ T cells, which was called immunologically the “cold” tumor microenvironment (Binnewies et al., 2018). Therefore, a better selection of patients who are most likely to benefit from immunotherapy is critical. In this study, we focus on the immune cell infiltration pattern difference between PDAC patients with different LNM status. We analyze the potential role of Th1 cells, Th1 cell–related genes in PDAC patients and further construct a prognostic gene signature–based risk score to predict overall survival and immunotherapy outcomes using bioinformatics models and machine learning methods.

## Materials and Methods

### Data Processing

The Cancer Genome Atlas (TCGA) mRNA expression data and copy number variation (CNV) information of samples were downloaded by the UCSC Xena browser (https://xenabrowser.net/datapages/), whereas the clinical information and mutation data were downloaded by using R package “cgdsr” and TCGAbiolinks, respectively.

In addition, we downloaded transcriptomic data of PACA-AU and PACA-CA from The International Cancer Genome Consortium (ICGC) to verify the relationship of immune cell infiltration ratio and prognosis. The expression data downloaded from the Gene Expression Omnibus (GEO) database (GSE85916) was used for validation of the risk model for prognosis. In addition, we used the R package to download the IMvigor210CoreBiologies data set for the prediction of immunotherapy outcomes. The data sets included in the analysis are summarized in Table 1 and detailed information of patients in the TCGA database is shown in Supplementary Table S1.

**TABLE 1:** The information of data sets.

Dataset	Patients (n)	Usage
TCGA-PAAD	177	Detect the relationship between the proportion of immune cell infiltration and prognosis
PACA-AU	269	Validate the relationship between the proportion of immune cell infiltration and prognosis
PACA-CA	186	Validate the relationship between the proportion of immune cell infiltration and prognosis
GSE85916	80	Validate the risk model for prognosis
IMvigor210CoreBiologies	348	Prediction of immunotherapy outcome

Abbreviations: TCGA, The Cancer Genome Atlas; PAAD, Pancreatic adenocarcinoma

### Estimation of the Abundance of Immune Cell Populations and Implementation of Weighted Correlation Network Analysis

To estimate the differences in proportion of 28 kinds of immune cells infiltrating in tumors between PDAC patients with lymph node metastasis and without in the TCGA database, we used single-sample Gene Set Enrichment Analysis (ssGSEA) in R package “GSVA” (Barbie et al., 2009; Hackl et al., 2016) (Supplementary Table S2). Then, survival analysis of these differentially infiltrated immune cells was performed (Supplementary Table S3), and the correlation between the score of immune cell infiltration and the number of lymph node metastases was further evaluated. Finally, we selected the cell population (X cell) with the most significant difference for subsequent analysis.

Immune-related genes (IRGs) downloaded from the Immunology database and Analysis Portal (ImmPort, https://www.immport.org/) and InnateDB (http://www.innatedb.com) databases were applied as the input of weighted correlation network analysis (WGCNA) based on the TCGA, PACA-AU, and PACA-CA databases (Supplementary Table S4) (Bao et al., 2019). The module depicting the highest correlation was considered the most correlated with X cell density. Gene significance quantified the association of individual genes with X cell density, and module membership represented the correlation between module eigengenes and gene expression profiles. Genes were selected as the hub genes in the module when the module membership (*x*-axis) was greater than 0.7 and the gene significance (*y*-axis) was greater than 0.5. Survival analysis was performed using the R package “survival”. Cox regression analysis was used to determine the hazard ratio (HR). All genes in the module were subjected to Cox regression. Then, the genes that were significantly associated with the survival of PDAC patients in the module were identified as the X cell-related gene signature (*p* < .01). These identified genes were applied to gene ontology (GO) analysis with the “clusterProfiler” package (Yu et al., 2012) to elucidate the potential mechanism behind the gene signature. R software (version: 3.5.3) was used for all the analyses in the manuscript.

### Prognostic Gene Signature–based Risk Score

The selected signature genes in the WGCNA module were analyzed with univariate Cox regression analysis. The comprehensive X cell–related signature was calculated by principal component analysis (PCA). The PCA based risk score “Cellpca” was derived from the first plus second principal components of the signature genes from the X cell–related gene signature. The formula for the prognostic gene signature–based risk score model was established as follows:

$${\mathrm{Cell}}_{\mathrm{pca}}=\sum PC1+\sum PC2\;.$$

### Copy Number Variation Analysis

The Genomic Identification of Significant Targets in Cancer (GISTIC) method (Beroukhim et al., 2007) was used to detect the CNV of all samples from SNP6 CopyNumber segment data. All aberrations are assigned a G-score that considers the amplitude of the events as well as the frequency of their occurrence across samples. The false discovery rate (FDR) q-values are then calculated for the aberrant regions, and regions with q-values below 0.05 are considered significant. For each significant region, we identified a “peak region” and a “wide peak” (confidence level is 0.90). The analysis is carried out through the corresponding MutSigCV module in the online analytic tool GenePattern (Mermel et al., 2011).

### Statistical Analysis

The survival curve of the prognostic analysis was generated by the Kaplan–Meier method, and the statistical significance was tested by the log-rank test. The prognosis prediction of the risk model was evaluated by the receiver operating characteristic curve (ROC), and the area under the curve (AUC) was quantified by R package “pROC.” Then, the R package “maftools” is used to present the mutation landscape of the samples.

## Results

### Th1 Cell Abundance Varied in PDAC Patients with Distinct Lymph Node Metastatic Status and Associated with the Prognosis

**FIGURE 1:**
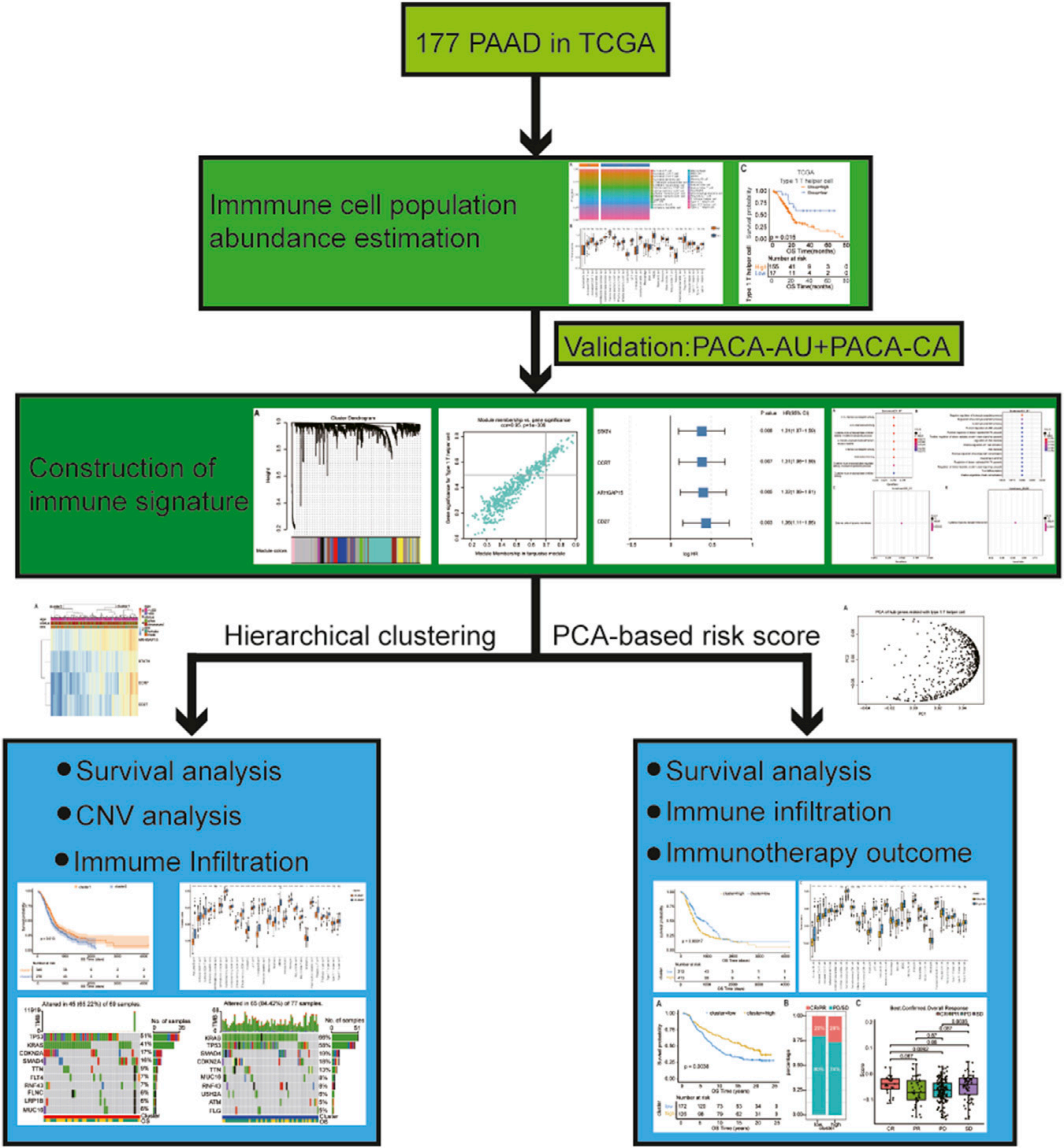
The workflow of this analysis. In brief, we compared the abundance of immune cell populations between PDAC patients with lymph node metastasis and without using ssGSEA. Then, survival analysis of these differential infiltrated immune cells was performed, and we selected the cell (X cell) with the most significant difference for subsequent analysis. After that, we screened the coexpression module depicting the highest correlation with the X cell infiltration ratio using WGCNA and further found out the hub genes associated with X cell density through univariate Cox regression analysis. We continue to analyzed the subtypes based on gene signature in two ways: 1) Subtypes obtained by hierarchical clustering analysis: analyzing the association of different subtypes with survival outcome, CNV, and proportion of immune cells infiltration; 2) Subtypes obtained by PCA-based risk score: constructing a risk model based on gene signature and then analyzing the association between different subtypes with survival outcome, CNV, proportion of immune cell infiltration and immunotherapy outcome, *etc*. Abbreviations: PAAD, pancreatic adenocarcinoma; PDAC, pancreatic ductal adenocarcinoma; ssGSEA, single-sample Gene Set Enrichment Analysis; WGCNA, weighted gene co-expression network analysis; CNV, copy number variation; PCA, principal component analysis.

**FIGURE 2:**
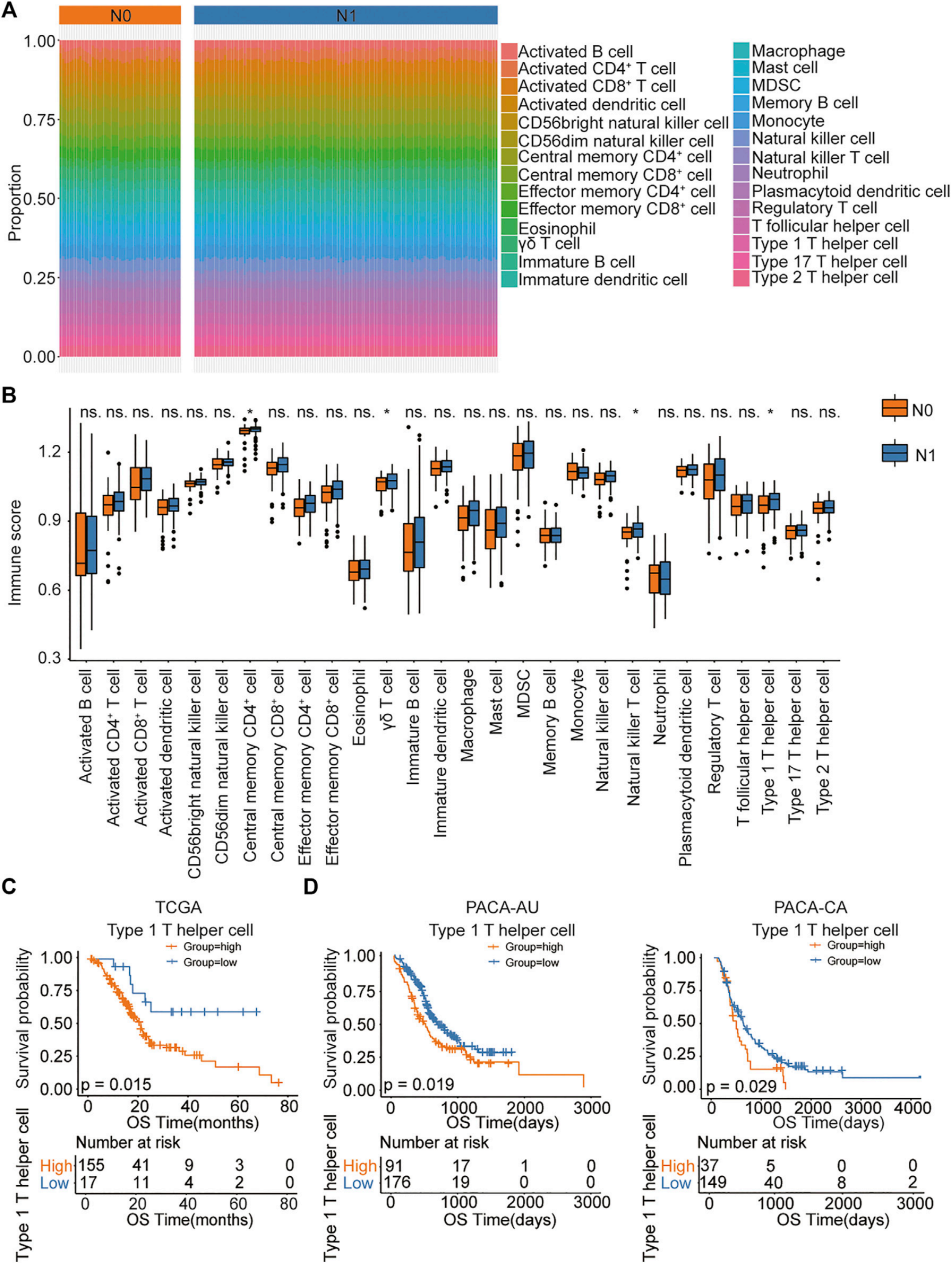
The association between immune cell abundance and lymph node metastatic status in PDAC patients. **(A)** ssGSEA analysis showing immune infiltration of 28 immune cells in TCGA pancreatic cancer samples. **(B)** The proportion of immune cell infiltration in lymph node metastatic and nonmetastatic samples. **(C,D)** Kaplan–Meier curves for the OS of PDAC patients showing that the patients with high type 1 T helper cell abundance had a poor outcome compared with the patients with low type 1 T helper cell abundance in the TCGA, PACA-AU, and PACA-CA cohorts. Abbreviations: CD, clusters of differentiation; MDSC, myeloid-derived suppressor cells; N0, indicate patients without lymph node metastasis; N1, indicate patients with lymph node metastasis; ns, no significant; OS, overall survival; PDAC, pancreatic ductal adenocarcinoma; ssGSEA, single-sample Gene Set Enrichment Analysis; TCGA, The Cancer Genome Atlas.

The workflow of this study ias shown in Figure 1. As reported in a recent article (Honselmann et al., 2020), we validated that PDAC patients with lymph node metastasis had worse prognosis (Supplementary Figure S1). To identify which immune cell type most correlated with the lymph node metastatic status in PDAC patients, we first analyzed the abundance of immune cell populations in tumor samples using ssGSEA based on the TCGA data set (Figure 2A). We identified 28 immune cell populations, and the correlations between lymph node metastatic status and these populations is shown in Figure 2B. We found that patients with lymph node metastasis had higher infiltration rates of type 1 T helper cells, central memory CD4^+^ T cells, γδ T cells, and natural killer T cell (*p* = .0442, .0479, .0353, and .0370, respectively) (Supplementary Table S2). Then, the association between the densities of these four types of immune cells and patient overall survival was evaluated based on the TCGA data set. The lower abundance of natural killer T cells, γδ T cells, and type 1 T helper cells benefited the survival of PDAC patients in the TCGA cohort (*p* = .019, .017, and .015, respectively; Supplementary Table S3; Figure 2C). We further compared the association between the score of immune cell infiltration and the number of lymph node metastases (Cancer Genome Atlas Research Network, 2017) (Supplementary Figure S2). Although the *p* values were all greater than .05, type 1 T helper cells had the smallest *p* value and the biggest Spearman correlation coefficient. Thus, in the present study, we chose type 1 T helper cells as the research objective. To further confirm the association between type 1 T helper cells and the survival of PDAC patients, we estimated the abundance of type 1 T helper cells in external cohorts (PACA-CA and PACA-AU). The results show that lower type 1 T helper cell abundance was also associated with prolonged survival of PDAC patients in these two databases (Figure 2D) as we observed in the TCGA-PAAD cohort. These results indicate that PDAC patients with lymph node metastasis tend to have a high proportion of type 1 T helper cells, and high abundance of Th1 cells is associated with worse prognosis.

### Identification of a Gene Signature Associated with Type 1 T Helper Cells

**FIGURE 3:**
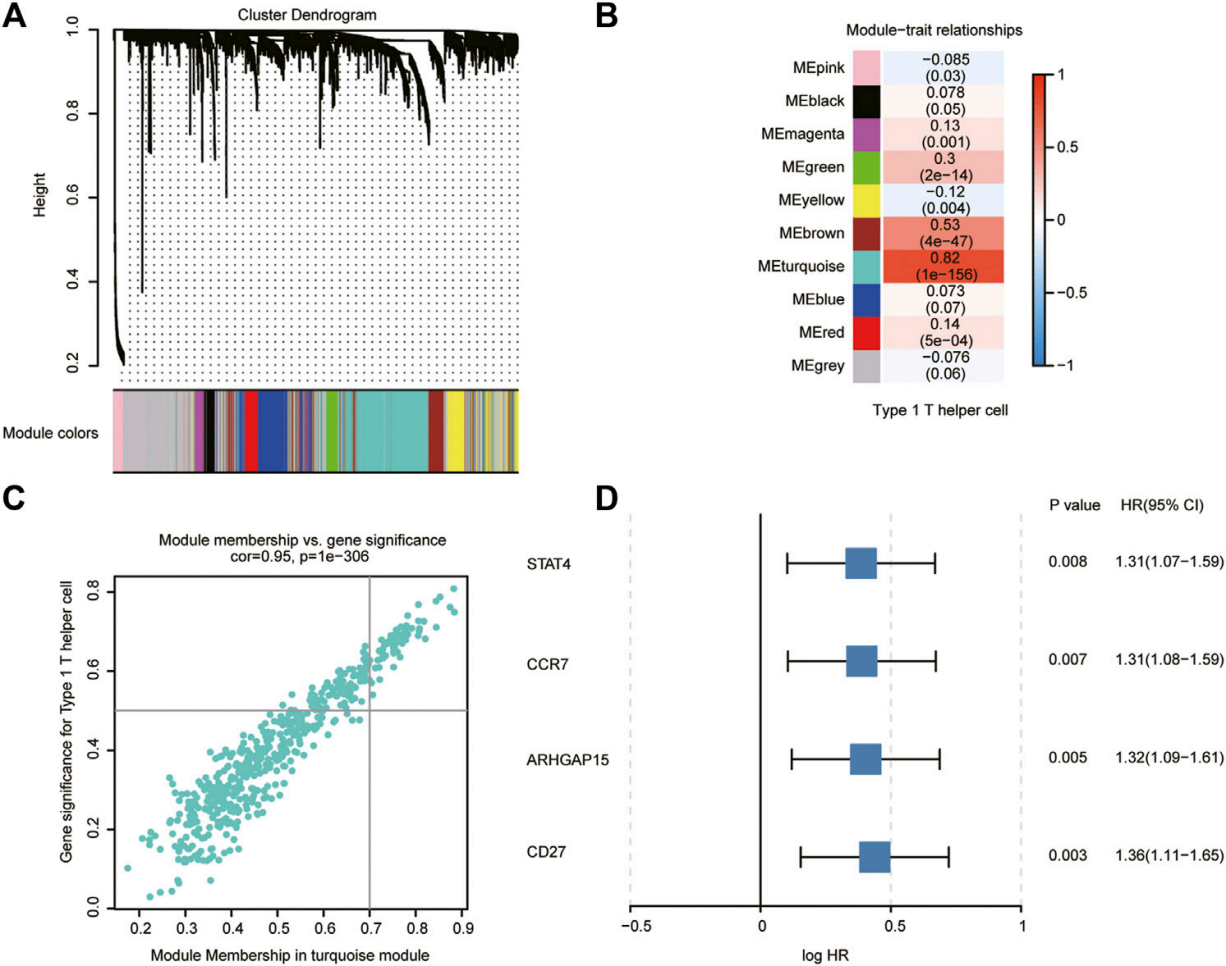
Type 1 T helper cell–related gene signature identification. **(A)** WGCNA was performed to identify 10 modules by unsupervised clustering. **(B)** A total of nine modules (nongray) were identified. The turquoise module had the highest correlation (r = 0.82, P = 1e^-156^) and was considered the most correlated with type 1 T helper cells. **(C)** The gene significance and module membership of the genes in the turquoise module exhibited a high correlation. **(D)** A total of four type 1 T helper cell–related genes that were significantly associated with the prognosis of PDAC patients were identified among the hub genes extracted from the turquoise module. Abbreviations: WGCNA, weighted gene co-expression network analysis; PDAC, pancreatic ductal adenocarcinoma; HR, hazard ratio; CI, confidence interval.

We applied WGCNA to identify modules of co-expressed genes that were enriched for association with type 1 T helper cells in the TCGA, PACA-AU, and PACA-CA databases. The immune-related genes obtained from the Immport and InnateDB databases were set as the input of WGCNA (Supplementary Table S4). Genes were clustered into 10 modules (Figure 3A). The correlation between the modules and type 1 T helper cell abundance was calculated by Pearson’s correlation coefficient (Figure 3B). The turquoise module showed the highest correlation coefficient with type 1 T helper cells (r = 0.82). The plots of module membership and gene significance illustrated a significant correlation for each gene in the turquoise module (r = 0.95; Figure 3C). The 62 genes whose module memberships were greater than 0.7 and gene significances were greater than 0.5 were considered as the hub genes in the turquoise module. Then, each hub gene in the turquoise module was analyzed with a univariate Cox regression analysis (Supplementary Table S5). Finally, we identified four hub genes that were significantly associated with the prognosis of PDAC patients (Figure 3D). GO analysis revealed that several molecular functions, biological processes, and cytokine–cytokine receptor interaction pathways were associated with the type 1 T helper cell–related hub genes (Supplementary Table S3).

### Molecular Subtype Identification Based on the Type 1 T Helper Cell–Related Hub Genes in PDAC

**FIGURE 4:**
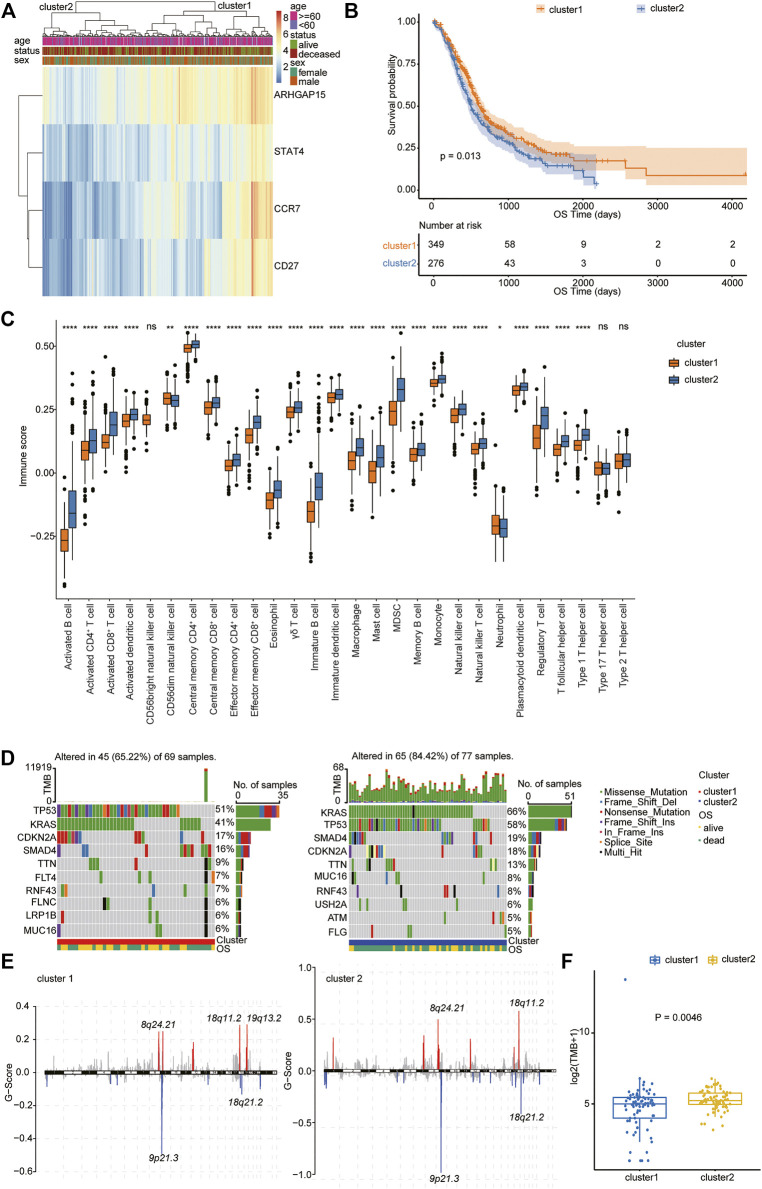
Molecular subtype identification according to the Type 1 T helper cell–related gene signature. **(A)** A hierarchical clustering based on four characteristic genes. **(B)** Kaplan–Meier curves for the OS of PDAC patients between clusters 1 and 2 in the TCGA, PACA-AU, and PACA-CA cohorts. **(C)** The proportion of immune cell infiltration between different clusters. **(D–E)** Distribution of gene mutations **(D)** and missing copies **(E)** between clusters 1 and 2 are shown, and tumor mutation burden is compared using Student’s *t*-test. Abbreviations: OS, overall survival; PDAC, pancreatic ductal adenocarcinoma; TCGA, The Cancer Genome Atlas; TMB, tumor mutation burden.

We then performed hierarchical clustering of transcriptional profiles of PDAC patients in the TCGA, PACA-AU, and PACA-CA databases based on the selected hub genes (Figure 4A) and divided the samples into two groups (clusters 1 and 2). We found that PDAC patients in cluster 1 had prolonged survival (Figure 4B). The immune cell population distribution in clusters 1 and 2 further illustrated the different tumor immune microenvironments in the two molecular subtypes of PDAC (Figure 4C). We then used the “maftool” package to summarize and analyze the mutational data in these patients. The common top 10 mutational genes in both groups are listed in Figure 4D. We find that KRAS, TP53, CDKN2A, SMAD4, and TTN were the common top five frequent mutational genes in both groups. To identify frequently changing areas, we used GISTIC 2.0 software to show the distribution of the occurring copy number variation region in the two sets of samples (Figure 4E). We found that the two clusters had significantly differences in tumor mutation burden (TMB) using Student’s *t*-test (Figure 4F). These results indicate that molecular subtypes of PDAC patients based on the selected hub genes show significant difference in prognosis, immune cell infiltration, and tumor mutation burden.

### Type 1 T Helper Cell–Related Signature Predicts the Prognosis and Clinical Outcome of PDAC Patients

**FIGURE 5:**
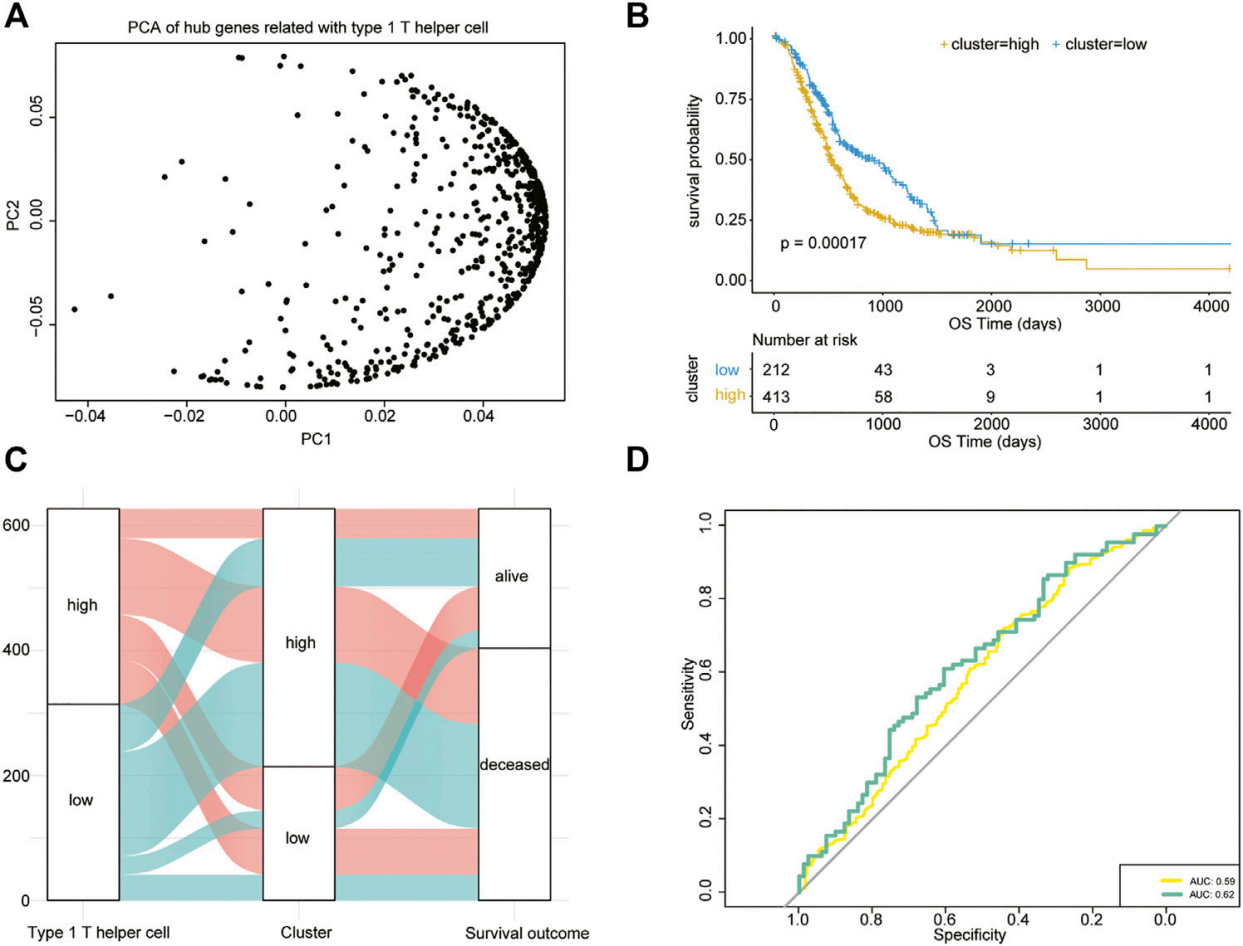
Type 1 T helper cell–related signature-based risk score calculation. **(A)** PCA of the key type 1 T helper cell–related genes. **(B)** Univariate Cox analysis and Kaplan–Meier curves show prolonged survival in patients with low risk scores compared with patients with high risk scores. **(C)** A Sankey plot is used to reveal the correlation between type 1 T helper cell scores, prognostic signature-based risk scores, and clinical outcome. **(D)** AUC estimation for the risk score model (yellow line) and incorporation of T stage into the risk score model (green line). Abbreviations: PCA, principal component analysis.

The type 1 T helper cell–related hub genes were employed to calculate a prognostic risk score. The risk score was calculated for each patient using the PCA method. Figure 5A shows the first (PC1) and second principal component (PC2) scores for hub genes related to type 1 T helper cells based on the TCGA, PACA-AU, and PACA-CA databases. The risk score of each patient is the sum of PC1 and PC2. The cutoff was determined using the “surv_cutpoint” function in the R package “survminer” and separated the whole PDAC cohort into high- and low-risk clusters (cutoff = 0.016) (Supplementary Table S6). The efficacy of the risk score model for predicting overall survival of PDAC patients was evaluated by performing Kaplan–Meier survival analysis. The result indicates that the risk score–based signature is significantly associated with the prognosis of PDAC patients (Figures 5B,C). One external cohort (GSE85916) was used to validate the association between the type 1 T helper cell–related gene signature and survival outcome in PDAC patients (Supplementary Figure S4). The ROC curve was used to evaluate the performance of this risk model in predicting outcomes, and the area under the ROC curve was 0.59 (Figure 5D). Incorporation of T stage into the risk score model has shown modest increase in the predictive performance (AUC = 0.62).

### The Association Between Risk Score Model and Gene Mutation, Immune Score in PDAC

**FIGURE 6:**
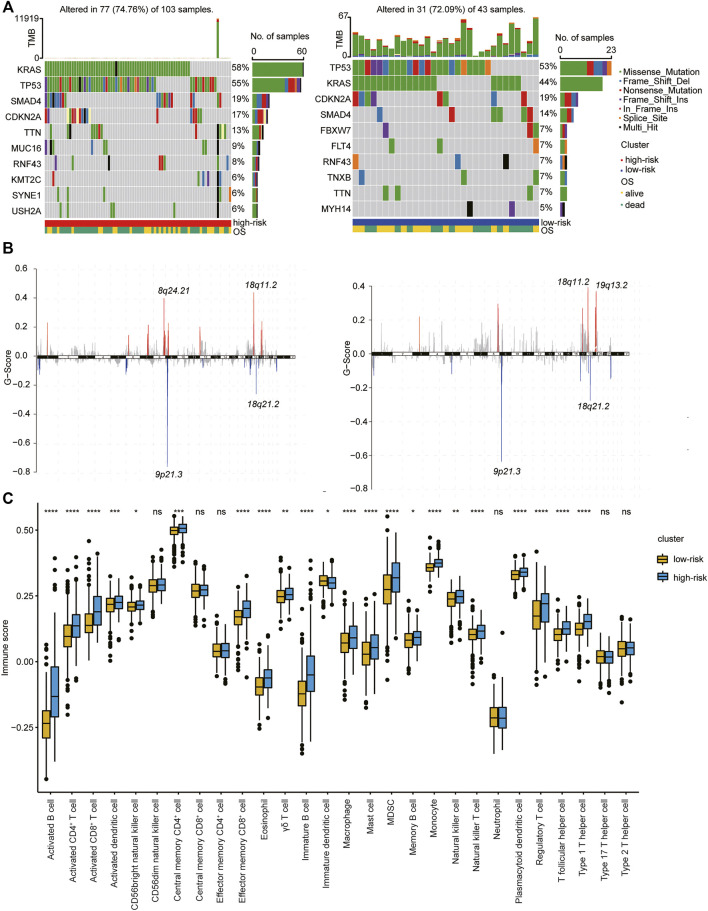
Association of type 1 T helper cell–related signature-based risk score with gene mutations in PDAC patients. **(A–B)** The distribution of gene mutations **(A)** and missing copies **(B)** between high-/low-risk score clusters (Left: high risk cluster; right: low risk cluster). **(C)** The proportion of immune cell infiltration between different clusters. *p*-value was calculated with Wilcoxon test. Abbreviations: OS, overall survival.

We then further explored the mutational data of patients in the high- and low-risk clusters using the “maftool” package. The common mutational genes in the top 10 of the two clusters and the distribution of the occurring copy number variation region in the two sets of samples are shown in Figures 6A,B. KRAS, TP53, CDKN2A, and SMAD4 were the common top four frequent mutational genes in both clusters. The immune cell population distribution in the high- and low-risk clusters further illustrates the different tumor immune microenvironments in the two subtypes of PDAC (Figure 6C).

### The Association Between the Risk Score Model and Immunotherapy Efficacy

**FIGURE 7:**
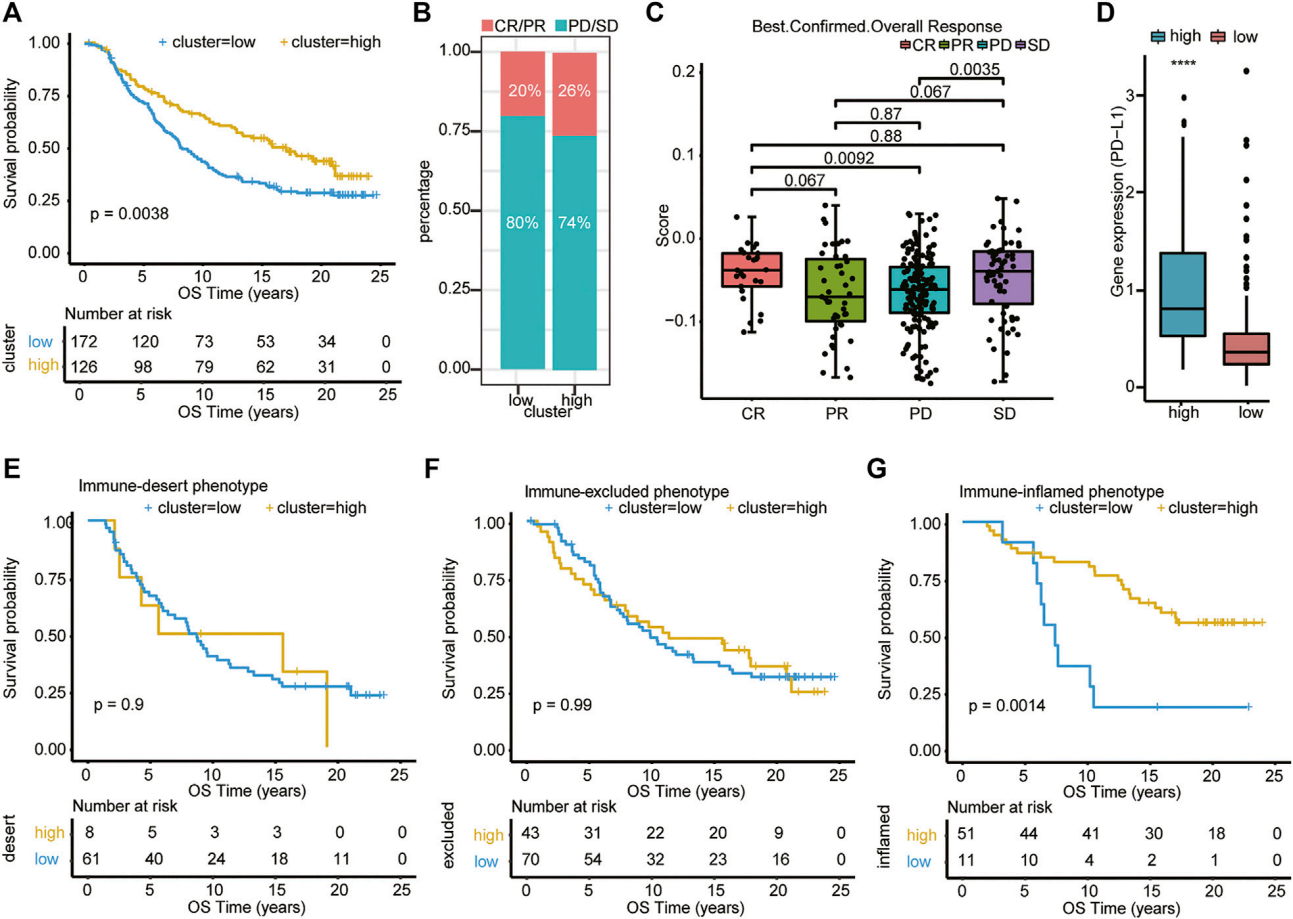
Association of type 1 T helper cell–related signature-based risk score with immunotherapy efficacy. **(A)** Univariate Cox analysis and Kaplan–Meier curves show that patients who received immunotherapy with high risk scores exhibited prolonged overall survival. **(B–C)** The distribution of immunotherapy responsiveness between two clusters was shown. *p*-value was calculated with one-way ANOVA test. **(D)** The PD-L1 expression level in the two clusters. **(E–G)** Kaplan–Meier curves show the survival difference in the immune subtypes, including desert, excluded, and inflamed subtypes between the high- and low-risk clusters within the IMvigro210 cohort. Abbreviations: CR, complete remission; OS, overall survival; PD, progressive disease; PDAC, pancreatic ductal adenocarcinoma; PR, partial remission; SD, stable disease; PD-L1, programmed death-ligand 1; OS, overall survival.

We subsequently tested the association between the risk score model and immunotherapeutic responsiveness using transcriptional expression information from the R package “IMvigor210CoreBiologies” (348 samples with 298 with immunotherapy outcome data). The cutoff was determined using the surv_cutpoint function in the R package “survminer,” and 298 samples were divided into high- and low-risk clusters according to the best threshold (cutoff = −0.04838594). Intriguingly, subsequent survival analysis showed that, among all patients who were treated with immunotherapy, patients in the high-risk cluster had significantly better OS (*p* = 0.0038; Figure 7A). Furthermore, out of 126 patients of the high-risk cluster, the best overall (confirmed) response was 26%, who reached CR/PR, and in patients of the low-risk cluster, only 20% were able to reach CR/PR (Figure 7B). Patients who reached CR or SD had a significantly higher risk score than patients in the PD groups (*p* = .0092 and .0035, respectively) (Figure 7C). Surprisingly, patients with PR seemed to have the lowest score of all four groups, but these differences (CR vs. PR, SD vs. PR, and PD vs. PR) were not significant. We then examined the PD-L1 expression in the two clusters and found that the high-risk cluster had a higher expression level of PD-L1 (Figure 7D). This may improve the therapeutic efficacy of immunotherapy in this cluster to some extent. We further analyzed the survival difference in the immune subtypes, including desert, excluded, and inflamed subtypes between the high- and low-risk groups within the IMvigro210 cohort. We found that patients in the inflamed subtypes had the most significant difference in survival analysis between different clusters (Figures 7E–G). These results indicate that patients in the high-risk score group may have better outcomes in the treatment of immune checkpoint inhibitors.

## Discussion

The PDAC is highly malignant with very poor prognosis. The fact that lymph node metastasis is an important related factor for poor prognosis in PDAC patients prompted us to search for the underlying molecular mechanism. Accurate prediction of prognosis can distinguish patients benefiting from the following treatment, such as immunotherapy. In this study, after obtaining resources from the databases, we first successfully identified that infiltration of type 1 T helper cells was highly associated with lymph node metastatic status and prognosis. We further found the turquoise module and four genes that were significantly related to the infiltration of type 1 T helper cells and prognosis. Subsequently, we classified these patients into two groups based on the gene signature and further constructed a risk score model based on PCA. We then explored their relationship with gene mutation, immune score, and clinical outcomes.

Regarding the impact of type 1 T helper cells on PDAC patient outcomes, numerous contradictory studies are reported. Many studies demonstrate that Th1 cells have a key role in combating cancers by secreting IFN-γ, IL-2, and TNF-α (Basu et al., 2021). However, a clinical trial shows that tumor progression may be promoted upon administration of IFN-γ (Alberts et al., 2008). Thus, Th1 cells may have multiple functions in tumor progression. Understanding the potential mechanism and roles of type 1 T helper cells in PDAC may be helpful for the development of immunotherapy. In our analysis, we reveal that a high abundance of Th1 cells is associated with shortened survival in PDAC patients. One possible reason for the differences and controversial conclusions in different studies may be the mixture of subtypes of Th1 cells in tumor tissues (Maggi et al., 2012). Laura Maggi et al. find that Th17 cells may shift toward Th1 cells in the presence of IL-12, and these cells were termed nonclassic Th1 cells (Maggi et al., 2012). The function of different subsets of Th1 cells may be varied. Therefore, it is essential to analyze the relationship of prognosis with different subsets of Th1 cells separately in the future.

In our study, STAT4, CCR7, ARHGAP15, and CD27 were selected as the hub genes to distinguish the different subtypes of PDAC patients. In general, these four genes are all related to the development, maturation, or function of Th1 cells (Oestreich and Weinmann, 2012; Margraf et al., 2020; Salem et al., 2021; Flieswasser et al., 2022). They may affect the prognosis of PDAC patients in many aspects. Previous studies show that high expression levels of the STAT4 protein may play distinct roles in different cancers (Cheng et al., 2015; Wang et al., 2015; Nishi et al., 2017; Zhao et al., 2017). However, the exact role of STAT4 in pancreatic cancer remains unclear. Similarly, high expression of CCR7 are shown to correlate with lymph node metastasis in pancreatic (Nakata et al., 2008; Guo et al., 2013) and many other cancers (Maekawa et al., 2008). However, the CCR7 axis is also involved in immune modulation and can assist in immunotherapy of cancers by potentiating the immune response to tumors (Förster et al., 2008; Nguyen et al., 2017). ARHGAP15 is shown to be related to better prognosis of early stage pancreatic cancer (Liao et al., 2017) and breast cancer (Takagi et al., 2018). CD27 is the receptor of the immune checkpoint molecule CD70 and is generally found on naive T, memory B, and T cell populations and subsets of NK cells. Dysregulation of the CD70-CD27 axis within the tumor and its microenvironment is commonly associated with tumor progression and immunosuppression (Jacobs et al., 2015).

KRAS was found to be the most significant mutation in PDAC patients who had worse prognosis in molecular subtypes and the prognostic gene signature–based risk score model. Our result is consistent with a previously published article (Chung et al., 2017). This result indicates the vital role of KRAS mutation in the development of PDAC. There is growing evidence that mutant KRAS drives the establishment of the immunosuppressive microenvironment in PDAC (Collins et al., 2012). Mutant KRAS could coordinate a paracrine network to build a tumor microenvironment that is composed of suppressive immune cells, activated stromal cells, and desmoplasia (Dias Carvalho et al., 2018). Mutant KRAS (mKRAS) could also drive tumor-intrinsic granulocyte macrophage colony-stimulating factor (GM-CSF) and chemokine C-X-C motif ligand 1 (CXCL1) expression, promoting MDSC infiltration (Bayne et al., 2012). Therefore, targeting mKRAS signaling may promote antitumor immune responses.

In our study, despite poor prognosis for PDAC patients in the high-risk group, patients in the IMvigor210 trial with high risk score benefit more from the PD-L1 blockage therapy. One possible reason for this interesting phenomenon is that the high-risk group has more infiltrated Th1 cells (Figure 6C), and more secreted IFN-γ may increase the PD-L1 expression (Figure 6D) of tumor cells, impairing antitumor immunity (Abiko et al., 2015). Furthermore, it is reported that prolonged IFN-γ signaling may promote both PD-L1-dependent and -independent resistance to ICI therapy and to a combination of ICI with radiation (Benci et al., 2016). They find that IFN-γ could enhance the expression of ligands for multiple T cell inhibitory receptors, such as TIM3 and LAG-3 (Benci et al., 2016). These results indicate that exposure to IFN-γ led not only to PD-L1 expression in tumor cells, but also to PD-L1-independent immune escape mechanisms. A study by Ayers et al. demonstrates that tumors with a specific type of TME characterized by active IFN-γ signaling are predictive of a good prognosis to PD-1 blockade with pembrolizumab (Ayers et al., 2017). The authors show that a group of IFN-γ-responsive genes were necessary for clinical benefit in nine different cancer types (Ayers et al., 2017). Likewise, the higher expression levels of the IFN-γ-responsive genes appears to be vital for the clinical success of anti–CTLA-4 mAb as evidenced by the analysis of tumor specimens from melanoma patients treated with ipilimumab (Mo et al., 2018). In a sense, when therapeutic regimens incorporate ICI agents, the presence of IFN-γ could correlate with better response and overall survival (Karachaliou et al., 2018; Zhang et al., 2020). Furthermore, we found that patients with PR seemed to have the lowest score of all four groups (CR, PR, PD, and SD) although these differences were not significant. This invites further investigation to compare other factors affecting the risk score model.

A problem with the type 1 T helper cell–related signature of PDAC prognosis as shown is that only *in silico* analysis is performed. Further experimental studies are required to elucidate the biological functions underlying the type 1 T helper cell–related signature in PDAC. These findings need to be interpreted with care and require validation in larger, well-designed prospective population-based studies.

## Data Availability

Publicly available data sets were analyzed in this study. The data can be found here: TCGA database (https://portal.gdc.cancer.gov); GEO database (http://www.ncbi.nlm.nih.gov/geo/); IMvigor210CoreBiologies data set (http://researchpub.gene.com/IMvigor210CoreBiologies/); immport (https://www.immport.org/); InnateDB (http://www.innatedb.com); ICGC database (https://dcc.icgc.org/).
