# Supplementary material for: Type 1 T Helper Cell-Based Molecular Subtypes and Signature Are Associated with Clinical Outcome in Pancreatic Ductal Adenocarcinoma
Source: Front Cell Dev Biol. 2022 Apr 1;10:839893. doi: 10.3389/fcell.2022.839893 (PMC9011157; doi:10.3389/fcell.2022.839893)
Supplement: Supplementary file 3 [file Table1.docx]

Supplementary Table S1. The clinical information of patients from TCGA database

| Clinical Features | Classification | Number |
| --- | --- | --- |
| N |  |  |
|  | N0 | 49 |
|  | N1 | 123 |
|  | Nx or '' | 5 |
| Survival status |  |  |
|  | Dead | 92 |
|  | Alive | 85 |
| Age |  |  |
|  | >=60 | 123 |
|  | <60 | 54 |
| Sex |  |  |
|  | Female | 80 |
|  | Male | 97 |

Abbreviatons: TCGA, The Cancer Genome Atlas; N, regional lymph node metastasis; Nx, regional lymph nodes cannot be assessed; '', provide no information regarding lymph node metastasis.
