# Supplementary material for: Type 1 T Helper Cell-Based Molecular Subtypes and Signature Are Associated with Clinical Outcome in Pancreatic Ductal Adenocarcinoma
Source: Front Cell Dev Biol. 2022 Apr 1;10:839893. doi: 10.3389/fcell.2022.839893 (PMC9011157; doi:10.3389/fcell.2022.839893)
Supplement: Supplementary file 7 [file DataSheet1.DOCX]

Supplementary Material


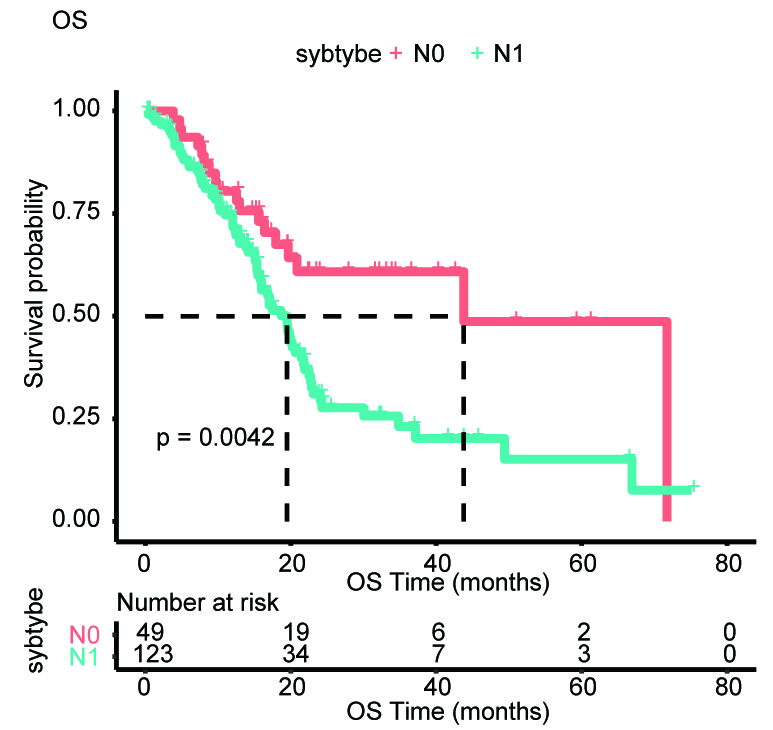


Supplementary Figure S1. Kaplan–Meier curves for the OS of PDAC patients in TCGA validate the association of lymph node metastatic status with prognosis. **Abbreviations**: OS, overall survival; PDAC, pancreatic ductal adenocarcinoma; TCGA, The Cancer **Genome** Atlas


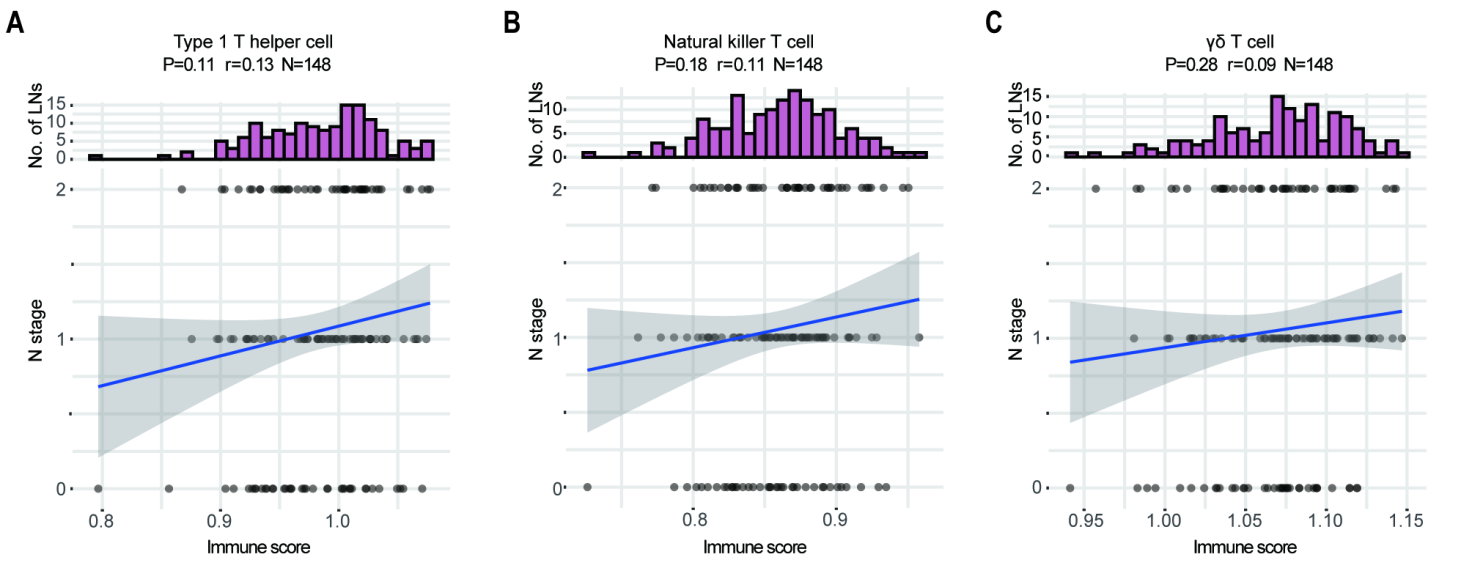


Supplementary Figure S2. The correlation between the score of immune cell infiltration and the number of lymph node metastases. (A) Type 1 T helper cell. (B) Natural kill T cell. (C) γδ T cell. Lymph node metastasis (N stage) were classified as follows: (1) N0: no regional lymph nodes metastasis; (2) N1: metastasis in 1-3 regional lymph nodes; (4) N2: metastasis in 4 or more regional lymph nodes.


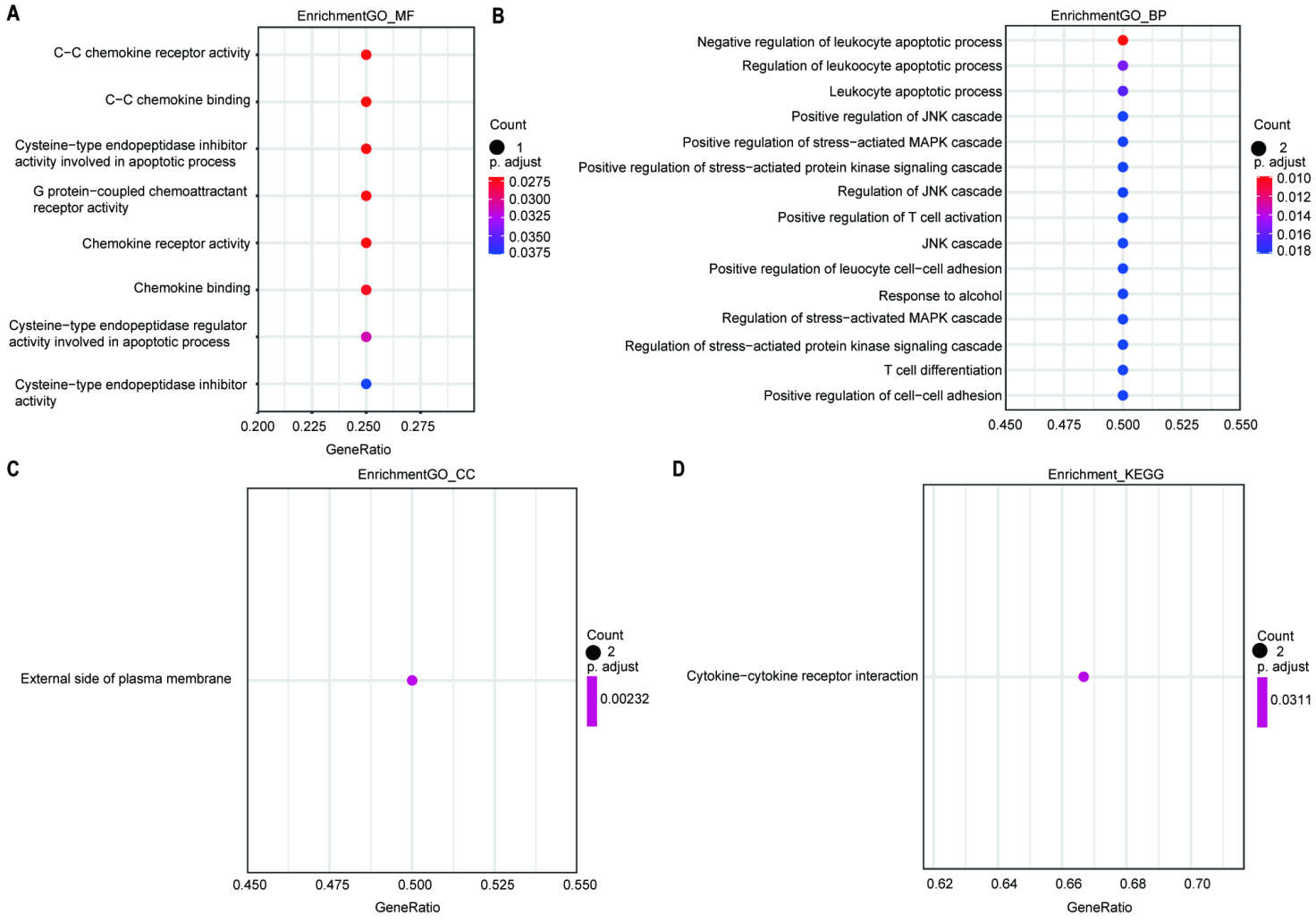


**Supplementary Figure S3.** GO and pathway enrichment analysis of selected hub genes (STAT4, CCR7, ARHGAP15 and CD27). (A) Top 8 enriched molecular functions of selected hub genes. (B) Top 15 enriched biological process of selected hub genes. (C) enriched cellular component of selected hub genes. (D) enriched KEGG pathways of selected hub genes. Abbreviations: GO, gene ontology; STAT, signal transducer and activator of transcription; CCR, C-C motif chemokine receptor; ARHGAP15, Rho-type GTPase-activating protein 15; CD, cluster of differentiation; KEGG, Kyoto Encyclopedia of Genes and Genomes.

**Supplementary Figure S4.** Kaplan–Meier curves for the OS of PDAC patients in GSE85916 validate the association of risk scores with prognosis.
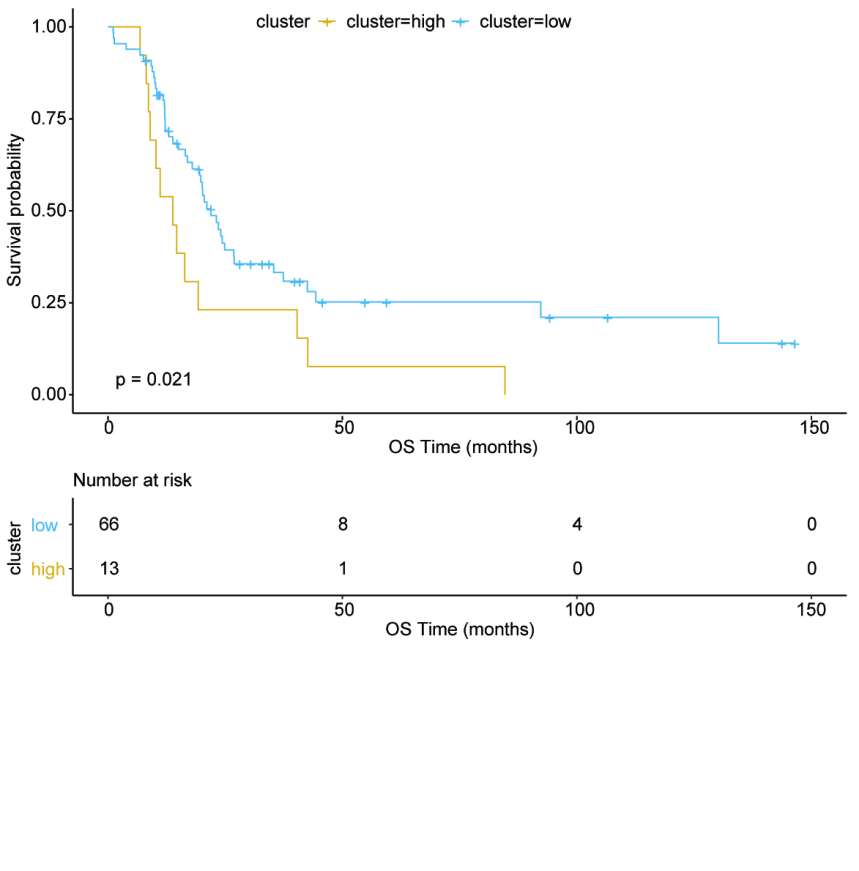
 **Abbreviations**: OS, overall survival; PDAC, pancreatic ductal adenocarcinoma.
